# Polyamines Interaction with Gaseous Signaling Molecules for Resilience Against Drought and Heat Stress in Plants

**DOI:** 10.3390/plants14020273

**Published:** 2025-01-18

**Authors:** Nidhi, Noushina Iqbal, Nafees A. Khan

**Affiliations:** 1 Department of Botany, Aligarh Muslim University, Aligarh 202002, India; nidhisharma0925@gmail.com; 2 Department of Botany, School of Chemical and Life Sciences, Jamia Hamdard, New Delhi 110062, India; naushina.iqbal@gmail.com

**Keywords:** abiotic stress tolerance, biosynthesis, ethylene, nitric oxide, hydrogen sulfide

## Abstract

Plants face a range of environmental stresses, such as heat and drought, that significantly reduce their growth, development, and yield. Plants have developed complex signaling networks to regulate physiological processes and improve their ability to withstand stress. The key regulators of plant stress responses include polyamines (PAs) and gaseous signaling molecules (GSM), such as hydrogen sulfide (H_2_S), nitric oxide (NO), methane (CH_4_), carbon monoxide (CO), carbon dioxide (CO_2_), and ethylene (ET). The functions of PAs and GSM in stress perception, signal transduction, and stress-responsive pathways have been explored. However, there is a lack of detailed, updated information on the interaction of PAs and GSM in the adaptation of drought and heat stress. This review explores the interaction between PAs and GSM for the adaptation to drought and heat stress. It explores their synergistic effects in mitigating the negative impacts of drought and heat stress on plant growth, development, and productivity. Moreover, a comprehensive analysis of physiological, biochemical, and molecular approaches demonstrates that their interaction activates key stress-responsive pathways, enhances antioxidant systems, and modulates gene expression. These combined effects contribute to improved drought and heat tolerance in plants. The information presented in the review provides valuable insights into plant stress resilience strategies and suggests potential measures for developing climate-resilient crops to address the increasing environmental challenges.

## 1. Introduction

For numerous years, it has been evident that unplanned and unintentional anthropogenic activities have been the primary contributor to the exacerbation of climate change, which has emerged as the most significant environmental peril. The enduring impacts of climate change on vegetation are inevitable. The issue of global food security is currently being plagued by the swift rise in population and significant alterations in the climate [1]. Due to the shifting climate patterns, drought and heat stress have emerged as the foremost impediments to crop yield and, by extension, food security [2]. According to the Food and Agriculture Organization, out of the 218.5 million hectares of cultivated wheat worldwide, 42% of the total area was affected by drought stress, while 58% of the total area was affected by heat stress [3]. Drought and high temperatures can potentially diminish crop productivity and yields, decreasing agricultural practitioners’ income. Based on the findings of the Intergovernmental Panel on Climate Change (IPPC) 2023 report, each increment of 0.5 °C (0.9 °F) in global temperature will result in a discernible escalation in the occurrence and intensity of heatwaves, heavy precipitation events, and localized droughts. Climate change significantly impacts the Earth’s crust, leading to irregular and sporadic precipitation patterns, heightened temperatures, and the expansion of areas affected by floods or water scarcity. These unfavorable circumstances contribute to the emergence of drought-prone regions, consequently affecting plant growth and crop productivity [4].

Furthermore, the extent of yield losses resulting from temperature and drought stress is subject to unpredictability. It depends on the annual precipitation pattern, surface water evapotranspiration, and soil water-holding capacity [5]. A recent investigation by the Indian Council of Agricultural Research (ICAR) (2021) found that the annual decline in agricultural yield in India, caused by drought and heat stress, exceeds a shocking ₹50,000 crore. Additionally, this study revealed a concerning prediction that the impact of these environmental stressors is expected to worsen in the future, mainly due to the adverse effects of climate change. The atmospheric concentration of greenhouse gases, namely carbon dioxide (CO_2_), methane (CH_4_), and nitrous oxide (NO), has experienced a notable increase, resulting in a net emission that has approached approximately 300 parts per million (ppm) in recent years [4].

The adverse effects of drought and heat stress on crop plants can be observed at the cellular and morphological levels. The severity of these effects depends on various factors, including species, genotype, developmental stage, cell type, organ, subcellular compartment, and the degree of water loss [6]. Drought stress occurs when the soil and atmospheric humidity levels are low and air temperature is high. Heat stress is the occurrence of elevated soil and air temperatures surpassing a specific threshold for a designated duration, resulting in irreversible damage to the growth and development of plants. An extensive study conducted across multiple locations emphasizes the influence of temperature on crop yields [4]. Plant heat stress leads to the perturbation of cellular physiological processes and alterations in molecular mechanisms [7]. When confronted with drought conditions, plants exhibit various adaptive responses, such as escape, avoidance, and tolerance. These mechanisms enable plants to cope with drought-induced stress effectively [4,8].

To cope with these adverse conditions imposed by heat and drought, plants have developed various mechanisms and strategies, and polyamines (PAs) are found to influence these strategies. PAs, including putrescine (Put), Spermidine (Spd), and spermine (Spm), are significant plant growth regulators (PGRs) that regulate plant development, senescence, and response to environmental stressors [9,10]. They are frequently found as independent molecular bases in nature. However, they can also be connected to small molecules such as phenolic acids in conjugated forms and different macromolecules like proteins in bound forms [11,12]. The prevalent conjugated PAs are those that are covalently bonded to cinnamic acids. The conjugation of PAs occurs by creating an amide linkage, utilizing esters of CoA to activate the carboxyl groups. PAs (Spd and Spm) control various plant growth and development processes, including root elongation, programmed cell death, membrane integrity preservation, and gene transcription. They also stimulate the antioxidant defense system by increasing the activity of antioxidant enzymes [13,14].

Along with PAs, gaseous signaling molecules (GSMs), such as nitric oxide (NO) and hydrogen sulfide (H_2_S), hydrogen peroxide (H_2_O_2_), CO_2_, ethylene (ET), and others, also protect the plant from various abiotic stress conditions. The involvement of many GSMs in plants under abiotic stress is an intricate and interrelated network. The GSM possesses a notable capacity to boost plants’ adaptive responses, which remains mostly unexplored in crop production, as per the latest study [15]. Every molecule has distinct activities, and their interactions contribute to plants’ overall response under stress. Plant scientists have shown remarkable interest in the biological roles of these GSMs, which were first identified as gasotransmitters in mammals [16]. The GSMs in plants include NO, CO_2_, H_2_S, ET, methane (CH_4_), and carbon monoxide (CO) [15]. The six criteria for identifying a GSM were previously presented, showing that CH_4_ is a gasotransmitter candidate comparable to H_2_S, CO, and NO. While review publications on the biology of abiotic stress linked to high-temperature and drought stress in plants are available, there has been no systematic investigation into the relationship between PAs and GSM-mediated abiotic stress in plants. The present review aims to synthesize the current information about the different functions of PAs and GSMs and their interaction in plant stress responses, focusing on how they contribute to the resilience of plants under drought and heat stress conditions. The PA and GSM interaction analyses may influence future research paths and strategies for improving plant stress tolerance in agricultural contexts.

## 2. Distribution and Biosynthesis of Polyamines

PAs are synthesized extensively in prokaryotes and eukaryotes, spanning more straightforward plant RNA viruses, phytoplanktons, and complex plants and animals [17]. PAs in higher plants are primarily found in an unbound condition. The Put, Spd, Spm, thermospermine (Tspm), and cadaverine (Cad) are the most often synthesized PAs in plants. In contrast, the remaining PAs are limited to certain plant species or environmental conditions [17]. It has been shown that immature leaves and apical meristems of tobacco (*Nicotiana tabacum*) plants have larger concentrations of the foremost PAs (Put and Spm) than old or mature leaves. However, the pattern of the Spd material was different. While Put is generated in hypogeous (underground) plant organs, the higher PAs (Spd and Spm) are primarily formed in aerial plant organs such as shoot apical meristems.

**Figure 1:**
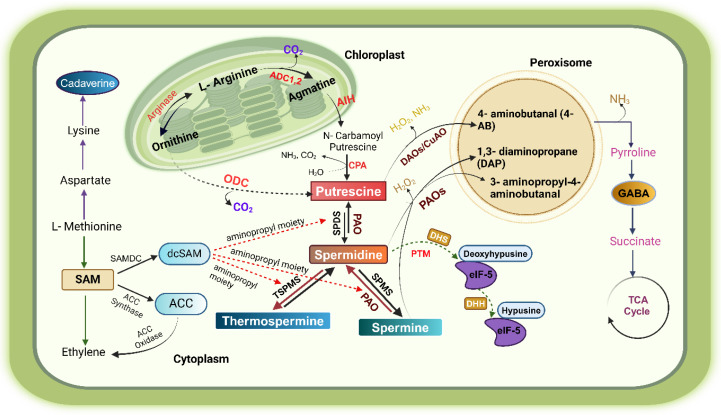
The diagram illustrates the biosynthesis route and catabolism of PAs in plants, depicting the enzymes involved in the metabolic process. Arginine decarboxylase 1,2 (ADC1,2), ornithine decarboxylase (ODC), agmatine iminohydrolase (AIH), N-carbamoylputrecine amidohydrolase (CPA), polyamine oxidase (PAO), diamine oxidase (DAO), spermidine synthase (SPDS), spermine synthase (SPMS), post-translational modification (PTM).

Several plant species have been investigated to understand the PA biosynthesis pathway [18,19]. In higher plants and bacteria, the initial step in PA biosynthesis is the decarboxylation of either ornithine or arginine, which is mediated by the enzymes ornithine decarboxylase (ODC) and arginine decarboxylase (ADC), respectively [20]. Agmatine is a byproduct of the arginine biosynthesis pathway of Put, which is then converted to diamine Put by the enzymes N-carbomyleputrescine amidohydrolase (NCPA) and agmatine imidohydrolase (AIH). In an alternate pathway, arginine (Arg) is first converted into citrulline (Cit) by the action of the enzyme arginine deiminase. Subsequently, the Cit undergoes decarboxylation by the catalytic action of citrulline decarboxylase (CDC), forming Put. Put is the central product of the PA biosynthetic pathway. The enzymatic addition of aminopropyl groups to Put from decarboxylated S-adenosylmethionine (dcSAM) yields triamine Spd and tetramine Spm [21]. SAM serves as a prevalent precursor for both PAs and ET. PA biosynthesis in plants and algae showcases remarkable diversity (Figure 1).

Moreover, akin to specific microbial thermophiles, they harbor a Spm unsymmetrical isomer known as Tspm, which actively participates in the development of plant vasculature [21,22,23]. Another pathway for Spd biosynthesis in grass peas has been identified, involving the conversion of aspartic acid or homoserine into aspartic semialdehyde, which combines with Put to form a Schiff’s base, reducing to carboxy-Spd and decarboxylated to Spd [17,24]. The lack of the *ODC* gene in Brassicaceae, including *Arabidopsis*, suggests that ornithine pathway-mediated PA synthesis is not crucial for typical development [17,25]. Spm is also present in plant nuclei and vacuoles, cell membranes, and other organelles. Additionally, there is a parallel biochemical route in which the amino acid methionine plays a role in converting Put to Spd and synthesizing Cad [26]. The study of polyamine biosynthesis provides the chance to modulate their concentrations and transport mechanisms that facilitate their distribution to various cellular compartments where they perform their biological roles.

## 3. Polyamine Metabolism in Plants

### Polyamines Catabolism

The levels of PAs are affected by the de novo synthesis and breakdown processes. The oxidation is primarily facilitated by specific enzymes called amine oxidases (AOs), copper-containing amine oxidases (CuAOs), and flavin-containing PA oxidases (PAOs) [27]. The importance of these molecules lies in their regulatory role in various physiological activities involving PA degradation. Additionally, their function is crucial in producing significant stress-related metabolites like 1,3-diamino propane (DAP), amino aldehydes, and H_2_O_2_ [26,28]. The Put content is regulated by two isoforms of the ADC gene (*ADC1* and *ADC2*) in *Arabidopsis*, even though their expression patterns are dissimilar [29]. In response to stress stimuli, the dynamic endogenous PA pool undergoes strategic interconversion, facilitated by various signal transduction pathways, the cellular compartmentalization of PAOs, other enzymes, and PA catabolism in response to elevated or decreased PA levels [30]. Recent studies have indicated that ABA-induced stomatal closure is the foremost drought and heat tolerance response mechanism. This response depends on reactive oxygen species (ROS)-related signaling, in which amino oxidases (AOs) and other PAOs play a significant role [31]. The oxidation of Spm by PAO3 leads to balanced respiration, which ultimately promotes transcriptional induction and helps maintain the balance of superoxide, H_2_O_2_, and reactive nitrogen species (RNS) levels [32]. The catabolism of PAs in plants commences with diamine oxidase (DAO) oxidizing Put to 4-aminobutanal, NH_3_, and H_2_O_2_ using Cu^+2^ and pyridoxal phosphate as cofactors. 4-aminobutal undergoes spontaneous cyclization to yield pyrroline (Pyrr). Subsequently, pyrroline dehydrogenase (Pyrr-DH) catalyzes the conversion of Pyrr into γ-aminobutyric acid (GABA). Following this, succinate semialdehyde is produced from GABA via GABA succinyltransferase. Subsequently, succinate dehydrogenase converts succinate semialdehyde to succinate, entering the Krebs cycle [17]. The catabolic enzymes for PAs are found in peroxisomes, cytoplasm, and mitochondria. PAOs are found in large amounts in monocots. PAO oxidizes Spm, which makes 1,3-diamino propane, H_2_O_2_, and 1-(3-aminopropyl)-4-aminobutanal. The latter spontaneously cyclizes to form 1,5-diazo bicyclo [4.3.0] nonane. When PAO reacts with Spd, it makes 1,3-diamino propane, H_2_O_2_, and 4-amino butanal. The 4-amino butanal then naturally cycles to make Pyrr [17,33] (Figure 1). *Arabidopsis* plants possess two enzymes, AtPAO1 and AtPAO4, which can convert Spm into Spd. On the other hand, AtPAO2 and AtPAO3 enzymes may turn Spm into Spd and, subsequently, into Put. In the false brome (*Brachypodium distachyon*), the BdPAO2 enzyme transforms Spm or Tspm into Spd and, subsequently, into Put. In contrast, the BdPAO3 enzyme only catalyzes the conversion of Spm to Spd [17]. Atanasov et al. [34] used the inherent diversity among *Arabidopsis* accessions to investigate the impact of guazatine, a fungicidal substance known to impede PA oxidases, on plant development. A specific location on chromosome 1 has been linked to guazatine tolerance. Within this location, a gene called *CHLOROPHYLLASE 1* (*CHL1*) has been identified as a potential candidate gene. PAs also play a crucial role in various cellular functions, including DNA replication, transcription, RNA modification, protein synthesis, ion channel regulation, antioxidant activity, cell cycle control, gene expression, and signal transduction [35]. The eIF5A protein undergoes a two-step post-translational modification of a specific lysine residue’s e-amino group, using Spd as the substrate. In this process, Spd supplies the 4-aminobutyl group needed to transform a conserved lysine in the translation elongation factor eIF5A into the atypical amino acid hypusine, which triggers factor activation [36]. This process is essential for the growth and proliferation of eukaryotic cells.

## 4. Advances in Genetic Manipulation of Polyamine Biosynthesis Pathways

Research conducted with various techniques such as RNAi, loss-of-function mutants and transgenic plants that overexpress specific genes or gene clusters, and stress-induced transcription factors has confirmed the protective roles of polyamines in drought tolerance. Using the RNAi technique, Belda-Palazón et al. [37] demonstrated that suppressing a gene responsible for encoding Arabidopsis deoxyhypusine synthase significantly impacts various facets of plant growth and development. Additionally, this suppression renders the plants more susceptible to stressors such as elevated salt levels, glucose, and exogenous ABA. The genetic manipulation of the polyamine biosynthesis pathway can significantly mitigate the damage caused by desiccation [38]. Several transcription factors have been identified as crucial for drought tolerance in rice (*Oryza sativa*). These transcription factors include the genes responsible for dehydration-responsive binding protein, APETALA type 2/ethylene-responsive factors, ABA-responsive element-binding protein 1, ABA-responsive binding factor 2, and myeloblastosis. These transcription factors regulate various functionally relevant protein kinases [39,40]. Overexpression of SPDS elevated several stress-induced transcription factors, producing stress-protective proteins (RD29A) [41]. Similarly, Spm transgenic plants demonstrated the ability to modulate gene expression associated with calcium regulation; the activation of mitogen-activated protein kinases; receptor-like kinases; and the biosynthesis of jasmonic acid, salicylic acid, and abscisic acid [42]. Research on the genetic modification of polyamine activity has elucidated how polyamines confer stress tolerance, particularly drought resistance, in plants. In a study, the constitutive expression of the SAMDC gene enhanced drought tolerance by decreasing reactive oxygen species production in Arabidopsis using the 35S::CaSAMDC construct [40]. The activity of PAOs was notably significant in both transgenic and wild-type plants. Additionally, the concentrations of ROS-detoxifying enzymes were significantly increased. However, the stress-induced synthesis of NADPH oxidase was markedly inhibited [43]. In addition to alterations in gene expression patterns, the overexpression of the stress-responsive transcription factor (PtsrMYB) improved the dehydration tolerance and markedly increased polyamine production via regulating the *ADC* gene [44]. Overexpression of PtADC in *Arabidopsis thaliana* ADC mutants resulted in increased drought tolerance and decreased ROS buildup. However, transgenic plants treated with ADC inhibitor D-arginine before stress induction have reduced ROS scavenging [45].

## 5. Transportation of Polyamines

Typically, cells can produce the necessary PAs via synthesis. Despite this, cells possess a very effective transport mechanism for absorbing external PAs. The existence of separate transport mechanisms for different PAs is uncertain. It is also unclear if a single transporter can transport all PA molecules. At the cellular level in higher plants, the absorption of PA is swift and becomes saturated within 1–2 min. This process follows a biphasic system with two components that may achieve saturation for Put [12]. The energy-dependent transport of PA across plasmalemma involves calcium (Ca^2+^) as a component of its mechanism [12,46]. Plant studies initially identified PA transport in apples (*Malus domestica*), and this biosynthetic compartmentalization implies that intracellular transport is critical for PAs’ physiological function [47]. PAs are predominantly localized in the vacuoles and cell walls of plant cells, with a lesser degree of presence in the chloroplasts and mitochondria [48]. Using various plant species, the mechanisms and kinetics of PA transport in protoplasts, vacuoles, and mitochondria have been investigated [46,47]. With a Km value of 61.8 M, the concentration-dependent transport of PAs into carrot vacuoles was biphasic, comprising linear and saturable phases [47]. Spm uptake was observed in mitochondria isolated from the tubers of Jerusalem artichoke (*Helianthus tuberosus*) with a Km value of 89 M [46]. Put was absorbed across the plasma membrane of maize (*Zea mays*) roots via a saturable, protein-mediated transport system with a Km value of 120 M [49]. Until recently, the properties of LAT family proteins in plants have remained unclear, but a study on *Arabidopsis* oxidative stress responses to the herbicide paraquat (PQ) has shed light on the role of a LAT family protein as a transporter of both PQ and PA [47]. Three out of the five *Arabidopsis* LATs demonstrating PA transport activity have diverse subcellular localization, indicating their participation in different cellular functions. For example, AtLAT1/PUT3 is linked to PQ resistance (e.g., RMV1 LAT1/PUT3) and is localized in the plasma membrane, suggesting a potential involvement in the intercellular transportation of PAs and PQ. In contrast, AtLAT3 and AtLAT4 were detected in the endoplasmic reticulum and Golgi apparatus [50]. Five more PA transporters, namely OsPUT2, OsPUT3, AtPUT1, AtPUT2, and AtPUT3, were discovered in rice and *Arabidopsis* by an experimental method [51]. The five PA transporters had affinities towards Put and Spd, as shown by their Km values of 28.7–33.4 μM for Put uptake and 0.94–3.3 μM for Spd [47]. The functions of these potential PA transporters are currently being identified, including the role of Organic Cation Transporter in Cad transportation and *AtLAT1*’s involvement in stabilizing mRNA during heat stress [52].

Research using sequencing data and yeast mutant complementation analysis found PA transporters in rice and *Arabidopsis* [51]. In a recent study, rice and Arabidopsis genes that exhibit sequence similarities to PA transporters were found in *Leishmania major* (*LmPOT1*) [53] and *Trypanosoma cruzi* (*TcPAT12*) [54].

## 6. Exploring the Potential of Polyamines for Heat and Drought Stress Tolerance

**Figure 2:**
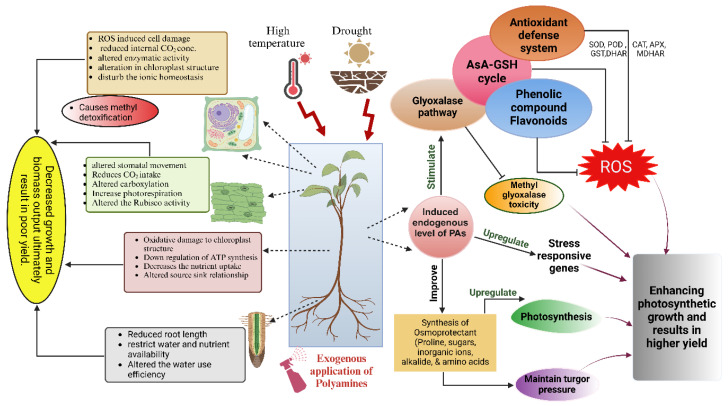
The illustration depicts the impacts of heat and drought stress on plants and the mitigating role of PAs. ASA-GSH, ascorbate-glutathione; APX, ascorbate peroxidase; CAT, catalase; DHAR, dihydroxy ascorbate reductase; GST, glutathione-s-transferase; MDHAR, monodehydroxy ascorbate reductase; PA, polyamine; POD, peroxidase.

PAs regulate plant defenses against abiotic stressors and normal growth and developmental processes [55]. A graphical presentation on the role of PAs in heat and drought stress is given in Figure 2. Heat and drought stress significantly impact plant growth, development, and productivity. These abiotic stressors frequently cooccur, exacerbating their detrimental effects on plants. Elevated temperatures can result in increased transpiration rates, the disruption of photosynthetic processes, and damage to cellular structures. Drought conditions further compound these issues by limiting the water availability, which is crucial for maintaining cellular turgor, nutrient transport, and various metabolic functions. The exogenous application of polyamines stimulates internal polyamine concentrations such as putrescine, spermidine, and spermine, playing a crucial role in alleviating heat and drought stress. These organic compounds function as osmoprotectants, maintaining the cell membrane integrity and stabilizing cellular structures under stress conditions. Polyamines also serve as antioxidants, scavenging reactive oxygen species (ROS) that accumulate during stress, thereby reducing oxidative damage. Additionally, polyamines modulate ion channels, enhance the photosynthetic efficiency, and regulate stress-responsive gene expression, improving plant tolerance to heat and drought stress.

### 6.1. Significance of Polyamines in Alleviating Heat Stress

Temperature stress substantially influences plant characteristics from seed germination to seed set through induced oxidative stress [56]. The synthesis of PA in Chinese kale leaves was dramatically increased by a high-temperature stress following a 6-day treatment. However, such an increase did not persist under extended exposure to heat. The content of the total PA and Put contents increased, but the increases did not persist for extended treatment periods. The total polyamines, including Spm, Spd, and Put, exhibited a steady rise, peaking after 9 days of therapy, followed by a subsequent decline [57,58]. The role of plant PAs is diverse, and the primary physiological mechanisms for withstanding high temperatures differ across plant species [58]. Studies have shown that wheat exhibits the greatest vulnerability to elevated temperature stress during grain filling, diminishing the grain weight and filling rate [59]. Consistent with prior studies, Wollenweber et al. [60] showed that high-temperature stress had a more pronounced inhibitory effect on inferior than superior grains. Earlier research has shown that rice can protect itself from the adverse effects of high temperatures by accumulating PAs when subjected to drought stress [61,62]. Jing et al. [62] showed that the exogenous application of Spm and Spd significantly enhanced the grain weight in superior and inferior grains under high-temperature stress. The endogenous Spd levels were positively correlated with grain weight, while the Put levels showed a negative correlation. The endogenous Spm content was positively correlated with grain weight but did not significantly correlate with the Spd concentration. The research determined that exogenous Spd and Spm significantly mitigated high-temperature stress damage during grain filling in wheat, mediated by external PAs. In tobacco plants, the early stage of heat stress increased the proline levels in the wild-type with a modest decline in the transformed variants. Nevertheless, following a 2-h delay, the concentrations of free and conjugated putrescine, free spermidine, norspermidine, and spermine increased, suggesting a reaction to heat stress and enhanced biosynthetic enzyme activity [63].

Another study by Cheng et al. [64] demonstrated that transgenic tomato (*Lycopersicum esculentum*) plants with genetic modifications, specifically overexpressing yeast SAMDC, exhibited significantly higher Spd and Spm levels than wild-type plants. This genetic alteration enhanced tolerance to heat stress by boosting the activities of antioxidant enzymes and safeguarding against membrane lipid peroxidation. Under physiological circumstances, the SPMS promoter (GUS construct) displayed increased activity in the roots, leaf edges, and vascular systems. However, when exposed to heat stress, the activity of the SPMS promoter was greatly amplified throughout the entire plant body. Intriguingly, the accumulation of GUS transcripts in this transgenic plant mirrored the behavior of endogenous SPMS transcripts under heat stress. Conversely, the levels of the *ACL5* transcripts remained unaffected by heat stress [19]. Amooaghaie et al. [65] proposed that PAs could potentially substitute Ca^2+^ under heat stress conditions in upholding cell membranes’ structural integrity. Heat stress resulted in the upregulation of two ADCs (SlADC1 and 2), one ODC (*SlODC2*), and two SAMDc genes (*SAMDc1* and *SAMDc3*) related to PA synthesis genes [66]. In rice plants, the exogenous application of Spd triggered an upsurge in the endogenous Spd and Spm levels. As a result, there was a subsequent increase in grain weight under heat stress conditions [67].

In response to an avirulent viral infection, Spm may trigger a particular signaling pathway by inducing the alternative oxidase (AOX) gene, which indicates mitochondrial malfunction [68]. The mitochondrial failure signal induces Heat shock protein-encoding genes [69]. Exogenous Spm may protect the plant from heat shock injury by activating various transcription factors, including *HsfA1a*, *HsfA1b*, *HsfA2*, and *HsfA3*. Recent research demonstrated that nearly all of the Spm-inducible genes previously identified in Arabidopsis could be induced by exogenously applied T-Spm [19], and in Arabidopsis HSF mutants, transcriptional and functional analyses revealed that *HsfA1a* and *HsfA1b* were essential for the initial phase of the heat stress response and that *HsfA2* regulates a subset of HSR genes, including ascorbate peroxidase 2, along a pathway distinct from that of *HsfA1a*/*HsfA1b* [70]. According to recent studies, non-canonical transcription factors have also been linked to the heat stress response. For example, heat stress activated the *bZip28* gene, which codes for membrane-tethered transcription factor, and the *bzip28* null mutant developed a hypersensitivity to HS [13,71].

### 6.2. Significance of Polyamines in Alleviating Drought Stress

The PA levels inside plant cells are crucial to counteract drought stress. The generation of H_2_O_2_ in guard cells plays a crucial role in ET-induced stomatal closure [72]. Putrescine works as a substrate and boosts ABA formation with the help of the intermediate activity of the calcium messenger to induce stomatal closure [73]. The lack of Spm increased drought sensitivity in the Arabidopsis mutant (acl5/spms), and the sensitivity could only be reverted by pretreating with Spm [26,74]. The exogenous spraying of Spd was found beneficial in the mitigation of drought stress [75]. Drought stress disrupts the balance of the production and elimination of ROS in plants, leading to the accumulation of free radicals of active oxygen, which hastens the oxidation of essential substances in the cell membrane, culminating in the formation of the lipid peroxidation product malondialdehyde (MDA) [76]. Nayyar and colleagues [77] found that the resilience of soybeans (*Glycine max*) to drought was improved with the exogenous administration of Put and Spd. Additionally, transgenic rice that had an increased expression of the *Datura stramonium ADC* gene showed greater resistance to drought as a result of the transformation of Put into Spd and Spm [78].

Moreover, spm enhanced drought tolerance in white clovers (*Trifolium repens*) by increasing the concentration of soluble carbohydrates (such as sucrose, fructose, and sorbitol) and decreasing the cytosolic Ca^2+^ levels [79]. Spm stimulates the upregulation of antioxidant enzymes, SOD, and CAT and boosts the glyoxalase system, resulting in a reduction of the stress-induced detrimental compound methylglyoxal [80]. The observation in *Lotus tenuis* of the transcriptional activation of stress-related genes (*AtADC* and *AtSPMS*) suggests that Put accumulation may have a cross-regulatory function in ABA production [9]. Transgenic lines that overexpress SAMDC or SPMS have shown increased gene expression due to Spm, which facilitates the triggering of transcription factors necessary for managing water scarcity [81]. Under drought stress, Tspm builds up and promotes metabolic and transcriptional reprogramming, increasing the sugar, proline, polyols, and TCA cycle intermediates. Additionally, there were changes in the expression of the genes related to auxin [26,82]. Cadaverine is a crucial precursor in forming secondary metabolites [83], and thus, exogenous cadaverine application modified the levels of Spm and the metabolism of PAs [30]. The exposure of Triticale (*Triticosecale wittm*) to drought during the late developmental stages resulted in a significant rise in PAs specific to the cell wall.

## 7. The Interaction of Polyamines with Gaseous Signaling Molecules

Studies have shown the interaction of PAs with GSM in the adaptation of abiotic stress. These are discussed in the following subheadings, and the selected studies on the interaction of PAs with GSM are given in Table 1.

### 7.1. Polyamines and Nitric Oxide

**Figure 3:**
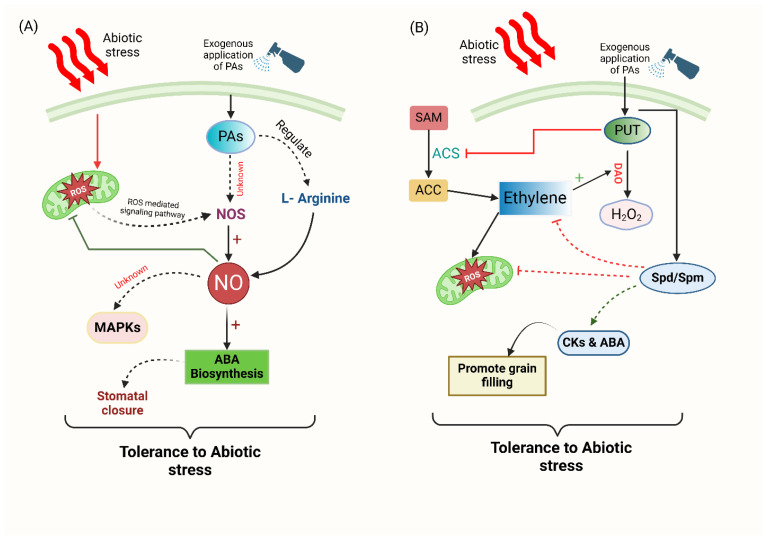
Illustration depicts the role of polyamines (PAs) in facilitating the signaling of nitric oxide (NO) and ethylene (ET) in plant cells. (**A**) Plant cells may produce NO via activating signaling pathways mediated by reactive oxygen species (ROS) catalyzed by an enzyme similar to nitric oxide synthase (NOS). PAs govern the potential synthesis of NO from L-arginine. The regulation of Mitogen-activated protein kinases (MAPKs) by NO depends on an elevation in the concentration of cytosolic Ca^+2^. NO also triggers stomatal closure by modulating the production of ABA, which enhances the plant’s ability to tolerate abiotic stress. (**B**) Polyamines (PAs) and ethylene may compete for S-adenosyl methionine (SAM), a common precursor for ethylene biosynthesis in higher plants, which is converted to 1-aminocyclopropane-1-carboxylate (ACC) through ACC synthase and oxidized to ethylene. Spermine (Spm) and other polyamines can control ethylene production by inhibiting ACC synthase, and ET boosts DAO production and function by oxidizing Put into H_2_O_2_. H_2_O_2_ reduces ET generation, preventing harmful effects on plants, and enhances PA generation by increasing ADC expression and ACD activity while preventing excessive ET production, which could negatively impact plants. Under drought stress, Spm treatment successfully reduced the generation of ET and ROS production while increasing the biosynthesis of cytokinins and ABA, which promote grain filling under stress conditions.

The interdependence between PAs and NO contributes to increased resilience against abiotic and biotic stressors. Arginine, an essential precursor for the biosynthesis of PAs, is also used to produce NO [91]. Studies further support the correlation between NO and distinct stress reactions triggered by PAs. Tun et al. [92] reported that PAs may interact with NO in the context of developmental and stress responses. This interaction was evident through the observation of NO production in response to the exogenous application of PAs [92,93] (Figure 3A). PA signaling-induced upregulation of NO production increases ABA biosynthesis and is crucial in tolerance to heat stress mechanisms [94]. Diamine oxidase (DAO) plays a crucial role in synthesizing H_2_O_2_ and NO, which are signaling mediators that can also significantly affect calcium signaling. It is reasonable to suggest that activating signaling cascades involving Ca^2+^, NO, and ROS as key components stimulates the protective systems responsible for developing heat resistance [95]. In the study of Wu et al. [96], mutants with reduced ornithine decarboxylase (ODC) activity and impaired Put production showed lower NO levels compared to the wild-type under heat stress. Exogenously applying Put elevated the endogenous Put in the ODC-silenced mutant when exposed to heat stress, resulting in a recovery of NO content to levels comparable to those of the wild-type. However, combining exogenous Put with carboxy-PTIO (NO scavenger) decreased the elevated NO content in the ODC-silenced mutants. The concentration of PAs and defense metabolites, such as proline and GABA, is sequentially enhanced by NO. This sequential relationship suggests an interplay between PAs and NO, understanding their involvement in a signaling crosstalk process.

### 7.2. Polyamines and Ethylene

It has been suggested that there is a connection between the biosynthesis pathways of SAM, Spd, Spm, and ET in plants. Externally applying PAs like Spd and Spm has been found to hinder ET production in different plant tissues. Moreover, ET has been shown to impede the functions of enzymes engaged in the synthesis of PA [58]. This indicates a potential communication or interaction among these biosynthesis pathways [97]. Given that ET and PAs share a common biosynthetic precursor known as SAM [88,98], it is entirely plausible that they interact in wheat grains in ways that regulate and influence the grain-filling process, ultimately affecting yield and quality (Figure 3B) [99]. These findings suggest that ET and PAs play a regulatory role in the process of grain filling. The variety of PAs can affect the ET levels, leading to varied outcomes. In drought conditions, wheat grain filling showed a decreased rate of ET release. The introduction of Spd and Spm during drought further reduced the rate of ET release, while the CKs and ABA levels increased, thereby improving wheat grain filling in drought conditions [100]. Furthermore, Huang et al. [101] found that drought caused a decrease in the activity of ribulose 1,5-bisphosphate carboxylase (Rubisco) in plants. Aminooxyacetic acid, an inhibitor of ET production, may potentially enhance the action of Rubisco by regulating the PA levels and mitigating the damage caused by ET release [102]. Additionally, the administration of Spm significantly reduced ET synthesis in nasturtium flowers that were subjected to drought stress. This reduction is attributed to the antagonistic concurrence between PAs and ET for the shared precursor, S-adenosylmethionine, indicating a complex interplay between these compounds under stress conditions [103]. Another study conducted by Talaat and Shawky [104] found that the application of Spm reduced ET production in maize (*Zea mays* L.) under drought stress. Young panicles exhibited a significant decrease in ACC content and ET evolution rate when treated with Spd and AVG (an ET inhibitor) while showing a substantial increase in free-Spd and free-Spm [105]. Conversely, the combination of ACC plus Methylglyoxal bis guanylhydrazone (MGBG), a potent SAMDC inhibitor, yielded opposite results. This study indicates that a metabolic interaction or competition between free-PA and ET biosynthesis may affect spikelet growth in rice under drought conditions [14].

Multiple researchers have explored the link between ET and PAs in plants under various abiotic and biotic stress conditions. ET plays a crucial role in fruit ripening and communicating with compounds like PAs to activate signaling against pathogens. An intriguing example is the interaction between ET, ROS, and PAs in peaches (*Prunus persica*) that have been affected by the destructive fungus *Monilinia* spp. PAs inhibit the synthesis of ET in a manner that is dependent on their molecular size, thereby retarding the onset of senescence [106]. Spermine is reported as the most potent inhibitor of ACC synthase [107].

Mehta et al. [108] found that the rate of ET synthesis in transgenic tomatoes carrying the yeast S-adenosylmethionine decarboxylase gene (*ySAMdc*) may increase up to three times the Spd/Spm compared to the rate of ET production in azygous control fruits. Additionally, in *Glycyrrhiza inflata* plants under stress, ET stimulated the activity of diamine oxidase (DAO) and PA oxidase (PAO). Additionally, it has been found that ET and ROS mutually reinforce productivity in plant cells through a complex interplay [109]. According to the transcriptome data analysis, it has been observed that PAs may have the capability to enhance ET production by upregulating ACS expression. Moreover, in transgenic tomato fruit with increased Spd/Spm accumulation, there was also an upregulation of S-Adenosylmethionine Synthetase (MAT) and Mitogen-Activated Protein Kinase Kinase (MAPKK) [110].

### 7.3. Polyamines and Hydrogen Sulfide

H_2_S can harm the plant when it is present in high concentrations and combined with abiotic stresses such as high temperature, drought, or salinity [111]. Nevertheless, at lower concentrations (10–1000 μmol L^−1^), it plays a role in signaling alongside other small reactive molecules like H_2_O_2_, NO, and CO [112].

In a study conducted by Chen et al. [113], it was observed that applying NaHS under drought conditions increased the biosynthesis of free and conjugated PAs. Hydrogen sulfide can potentially enhance plant growth and photosynthesis in the presence of drought-induced stress. It increases the transcription levels of *SoADC*, *SoCPA*, *SoODC*, and *SoSPDS* during drought conditions, leading to the enhanced production and accumulation of PAs in plant tissues. This can improve resilience against environmental challenges [114]. Li et al. [115] found that drought increased the H_2_S content and L/DCD (L/D cysteine desulfhydrate) activities, which dicychlohexylamine (DCHA) reversed. Exogenous Spd alleviated the inhibitory effect of DCHA on H_2_S production and L/DCD activities under dehydration conditions, indicating Spd’s role in dehydration-induced H_2_S signaling in white clovers. The recent study by Li et al. [115,116] indicated that Spd can activate the L/DCD pathway, which leads to H_2_S signaling. Furthermore, signal transduction regulates transcription factors (such as bZIP37, bZIP107, DREB2, DREB4, and WRKY108715) that play a role in white clover leaves’ drought response and antioxidant defense. The control of DREB2 protein by Spd via H_2_S signaling could be critical for the leaf tissue’s response to drought stress.

**Figure 4:**
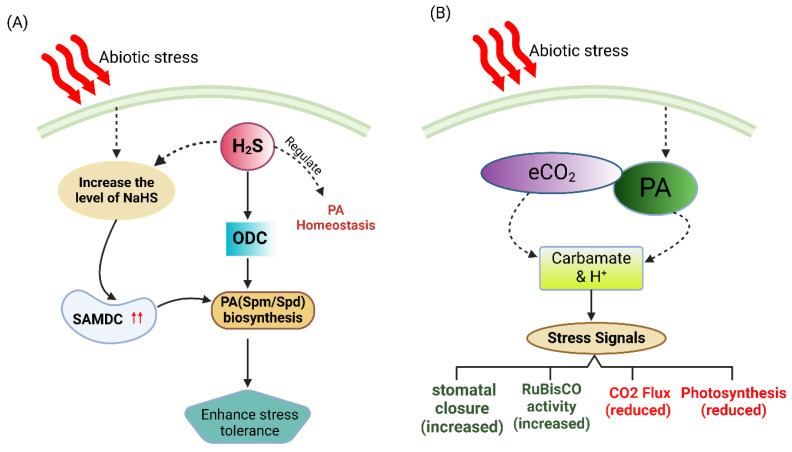
An illustrative hypothetical diagram depicts the interaction between PAs and H_2_S and the levels of CO_2_. (**A**) When plants are experiencing drought stress, the use of NaHS increases the activity of the genes responsible for producing polyamines, specifically arginine decarboxylase (ADC) and ornithine decarboxylase (ODC). As a result, the levels of polyamines in the plant tissues increase. Increased levels of NaHS may potentiate the impact of polyamines by further increasing the expression and activity of SAMDC. The synergistic impact may enhance the plant’s capacity to withstand stress. (**B**) Polyamines (PAs) contain multiple amine groups that can react with carbon dioxide (CO_2_). Under stress conditions, the concentration of CO_2_ in the plant’s intercellular spaces increases, allowing it to respond with PAs. This reaction leads to carbamate and hydrogen ions (H^+^) formation. Carbamate formation can be part of the plant’s stress response mechanisms, such as inducing stomatal closure and enhancing the activity of Rubisco (the enzyme responsible for carbon fixation in photosynthesis) while reducing the influx of CO_2_, decreasing photosynthesis.

When plants experience drought stress, the use of NaHS has two effects. Firstly, it increases the activity of the genes responsible for producing PAs, specifically arginine decarboxylase (ADC), ornithine decarboxylase (ODC), and N-carbamoyl putrescine amidohydrolase. Secondly, it decreases the activity of S-adenosyl-met-decarboxylase (*SAMDC*). This leads to higher levels of PAs in the plant tissues [117]. Further research is required to elucidate the precise mechanism by which H_2_S mediates the synthesis of PAs in response to abiotic stress (Figure 4A).

### 7.4. Polyamines and Carbon Dioxide

Carbon dioxide is a colorless and odorless gas that naturally exists in the Earth’s atmosphere, playing a crucial role in the planet’s carbon cycle, and is essential for photosynthesis in plants. Carbon dioxide is essential for signaling pathways in plants during stress. It triggers the production of ROS and activates stress-related genes, regulating the plant’s response to environmental challenges like drought, high temperatures, and pollutants [118].

Extreme high temperatures may cause drought stress, which, in turn, reduces stomatal conductance and causes a significant rise in the intercellular CO_2_ concentration. This ultimately leads to a drop in the photosynthetic activity of plants [119]. Furthermore, the combined effect of heat stress and elevated levels of CO_2_ on PA metabolism, as well as plant physiology, is an area that warrants further investigation. When plants are exposed to high-temperature stress and CO_2_ levels, PA biosynthesis is inducted, increasing the accumulation of PAs in plant cells. This induction of PA biosynthesis in response to CO_2_ stress suggests that PAs may be involved in the plant’s defense and adaptation mechanisms against high CO_2_ levels [120]. The research conducted by Logothetis et al. [86] suggested that plant culture cultivated under high levels of CO_2_ had elevated levels of Put content, thereby affecting the Put/Spm ratio and influencing the size of light-harvesting chlorophyll a/b-binding protein complex II. This demonstrates the influence of CO_2_ on PA levels and its impact on plant photosynthetic machinery (Figure 4B). The specific molecular mechanisms underlying the CO_2_–PA interaction in plants under abiotic stress are still being explored.

### 7.5. Polyamines and Carbon Monoxide

**Figure 5:**
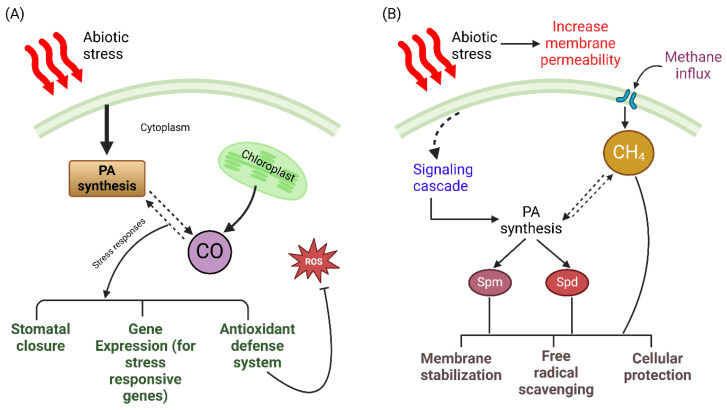
A hypothetical representation of the interaction of PAs with CO and CH_4_. (**A**) Under stress conditions, the generation of CO may increase substantially. While carbon monoxide may not directly interact with cytoplasmic PAs, it functions as a signal that initiates a series of actions resulting in the manufacture of PAs or changes in their activity. This process could involve other signaling molecules or changes in gene expression, and this interaction may activate the antioxidant defense system, which can help mitigate the effects of ROS activity and enhance heat stress tolerance. (**B**) Heat and drought stress can increase membrane permeability, disrupting cellular homeostasis. Methane may act as a signaling molecule in plants, triggering signaling pathways that increase the production of PA biosynthesis enzymes. PAs such as spermidine and spermine can improve membrane integrity, facilitate free radical scavenging, and help mitigate the detrimental effects of heat and drought stress on plant cells.

Carbon monoxide acts as a signaling molecule in plants, helping to alleviate oxidative stress caused by environmental factors such as high temperature, drought, water shortages, and heavy metals. This CO then plays a crucial role in regulating the plant’s responses to these abiotic stressors and functions crucially in signaling redox reactions inside cells and activating the antioxidant defense systems. Drought stress induces the production of ROS in plants [121]. CO is also described as a promoter of chlorophyll accumulation in plants under stress conditions. Chen et al. [113] reported that CO was crucial in increasing the chlorophyll levels in cucumber (*Cucumis sativus*) plants under drought conditions by interacting with hydrogen gas. This study indicates that CO may enhance the chlorophyll content in drought, and CO is an essential regulator of chlorophyll synthesis and plant resilience during stressful conditions (Figure 5A).

Carbon monoxide interacts with signaling molecules, NO, and H_2_S to modulate stress responses and bolster plant resilience against environmental challenges. The impact of gaseous CO on physiological processes in plants was discovered later. However, its role as a GSM in plant cells is not as well researched as NO and H_2_S [120]. The interaction of CO and PA in plants is not well studied. The topic is exciting for research, with great promise. Gaseous CO or exogenous hematin applied as a CO donor to aqueous solutions induces dose-dependent stomatal closure in *Vicia faba* [122,123].

### 7.6. Polyamines and Methane

Methane, a potent greenhouse gas, has been found to play a complex role in the response of plants to abiotic stress. Plant CH_4_ production has increased under abiotic stress conditions such as drought, salinity, and extreme temperatures. This increase in CH_4_ production is thought to be a response mechanism by which plants can alleviate the harmful effects of abiotic stress. Although the exact mechanism behind plant CH_4_ synthesis must be identified, ROS may be a significant hub for stimulating plant CH_4_ production [10]. Research has demonstrated that plants can autonomously produce CH_4_ in aerobic environments. Plants’ production of CH_4_ has been attributed to four distinct mechanisms. (a) CH_4_ is produced when sodium azide inhibits the electron transport chain in the inner membrane of plant mitochondria. (b) H_2_O_2_ oxidizes methionine to generate methionine sulfoxide. To generate methyl radicals, methionine sulfoxide can demethylate itself by homogenizing the split-cleavage bond that liberates CH_4_. (c) Amino acids combine to produce amino acid methyl when exposed to blue light. Oxygen species react with the amino acid methyl in canola to produce CH_4_. (d) When struck by ultraviolet radiation, tryptophan generates singlet oxygen (^1^O_2_) [123]. Additionally, in the presence of biological reductants, singlet oxygen (^1^O_2_) may transform into the reactive hydroxyl radical (·OH). Consequently, the reaction between ultraviolet radiation and tryptophan generated hydroxyl radical (·OH) and facilitated the generation of CH_4_ from the methyl group of the pectin ester [124].

It was discovered that, by modulating the expression of the heme oxygenase-1 (HO^−1^) gene, CH_4_ could reduce the excessive accumulation of ROS during the germination of alfalfa seeds, thus reducing the inhibition of seed germination. The interaction of CH_4_ with PAs under heat and drought conditions is yet to be discovered. PAs may indirectly influence CH_4_ production by modulating the stress response pathways that initiate methanogenesis (Figure 5B). In Han et al.’s [125] study, CH_4_ was shown to mitigate the effects of polyethylene glycol (PEG) stress, a solute that simulates water shortage and induces osmotic stress. This is achieved by modulating the ROS levels and improving the system’s balance of sugars, ascorbic acid (AsA), and glutathione (GSH). Abdulmajeed and Qadri [126] showed that higher temperatures and UVB radiation caused increased CH_4_ emissions from pea (*Pisum sativum*) plants. The emissions were most significant in the top section of the shoot and stem and the lower leaves.

Methane and PAs are both essential for plant signaling and adaptation. While PAs are widely recognized as regulators for mitigating heat and drought stress, the role of CH_4_ as a signaling molecule under these stress conditions is still being investigated. Understanding these processes could lead to novel approaches to enhance the resilience and productivity of crops.

Employing advanced molecular techniques can help us better understand how plants respond to polyamine signals. This can shed light on the interaction of PAs with other signaling molecules, supporting the development of stress-resilient crops. At the cellular level, many innovative methodologies are used to elucidate the processes of GSM and PA interactions. The key approaches include (i) Fluorescence Resonance Energy Transfer (FRET): this technology enables the construction of FRET-based biosensors to investigate interactions, (ii) Optogenetics: this method elucidates the real-time interaction between GSMs and PAs, establishing a signaling network inside cells, (iii) Genetically Encoded Sensors: these sensors can monitor changes in GSMs and PAs in reaction to external stimuli, (iv) Single-Cell RNA Sequencing: used to investigate the regulatory effects of GSMs and PAs on gene expression and cellular mechanisms, and (v) CRISPR-Cas9 and RNA Interface: it elucidates the processes of GSM and PA interactions at the genetic and protein levels. CRISPR technologies have demonstrated promise in agriculture, and next-generation CRISPR techniques offer improved accuracy. Inactivating Cas9 and Cpf1 nuclease domains enable the combination of DNA-targeting proteins with diverse enzymatic activities, facilitating the study of signaling pathways linked to GSM molecules.

## 8. Reactive Oxygen Species Mediate Polyamine and Gaseous Signaling Molecule Interactions

The mediation of the GSM and PA interactions largely depends on ROS homeostasis. The cellular responses to different external environmental conditions are regulated by the equilibrium of ROS, GSMs, and PAs. PAs encourage ROS generation. Spermidine and spermine affect the functioning of mitochondria, which causes an increase in the formation of ROS. In the same way, GSMs interact with ROS. They may either scavenge them or adjust how they are produced. The interconnections between ROS, GSMs, and PAs are important for cell proliferation and differentiation, as well as for cellular stress responses. When there is a lot of stress, the formation of ROS increases, which is important for regulating the activity of enzymes in GSM and PA metabolism and the redox state of the cell. Reactive oxygen species may either stimulate or inhibit spermine oxidase and polyamine oxidase, which affects the amounts of polyamines. On the other hand, ROS may affect the generation of NO, H_2_S, and CO via a variety of signaling mechanisms.

## 9. Identifying the Gaps in Understanding the Interaction of Polyamine and Gaseous Signaling Molecules

Despite progress in understanding how polyamines and GSMs improve plant drought and heat stress tolerance, numerous key gaps remain. The research should focus on understanding the complex interactions between PA-gas signaling pathways, uncovering the molecular mechanisms involved, and developing new strategies to enhance plant stress resilience. The effects of polyamines and GSMs on various plant species in different environments are also unknown. Studying the synergistic effects of polyamines and GSMs with phytohormones and signaling molecules may help explain plant stress tolerance. Advanced biotechnological methods to control polyamine and GSM pathways may help build stress-resistant crops. Sustainable agriculture in the face of climate change requires closing these gaps. With collective efforts, we can tap into the complete capabilities of PAs and GSMs, which serve as crucial elements for enhancing plant resilience amid a shifting climate and ever-changing environmental obstacles.

## 10. Conclusions and Future Perspective

The increasing severity of abiotic stresses, specifically heat and drought due to climate changes, poses significant threats to plant growth and crop productivity. By thoroughly examining the existing research, this review shed light on the crucial roles of PAs and GSMs in coordinating adaptive responses in plants when confronted with challenging environmental conditions. PAs, such as Put, Spd, and Spm, play a crucial role in the complex web of stress responses. PAs demonstrate adaptability as regulators of gene expression, controllers of enzymatic activities, and facilitators of cellular signaling pathways. These components are crucial to the plant’s defense system, helping it withstand various environmental challenges like drought and heat stress. In parallel, gaseous signaling molecules play a vital role in plant stress responses, impacting multiple aspects of development and tolerance to stress.

The PAs and GSMs work together or against each other to influence how plants respond to their surroundings. Although the exact molecular mechanisms behind this interaction are not yet fully understood, it is becoming increasingly apparent that PAs and GSMs work together to adjust plant stress responses finely. In addition, the complex interactions between PAs and GSMs highlight the possibility of selectively manipulating these pathways to improve crop stress tolerance. This, in turn, can contribute to sustainable agriculture amid increasing environmental challenges.

## Figures and Tables

**Table 1:** Selected studies on the interaction of polyamines with gaseous signaling molecules in plant adaptation to drought and heat stress.

S. NO.	Plant Species	Stress	Polyamine(s)	Gaseous Signaling Molecule	Interaction	References
1.	*A. thaliana*	Drought stress	PAs (Put, Spd, Spm) 0.5 mM	Induce NO synthesis	PAs significantly increased NO levels in stomatal guard cells, allowing for stomatal closure to ameliorate water scarcity.	[84]
2.	*Rosa damascena Mill.*	Water deficit stress (The treatments consisted of three irrigation levels: 25%, 50%, and 100% of field capacity).	The highest content of Spd and Spm were observed under moderate (25%) and severe drought stress (50%), respectively	The expression of NOS escalated from 3 to 12 days, exhibiting a 54-fold increase under 50% and 25% field capacity.	Increased the expression of NOS (nitric oxide synthase) and induced the stomatal closure	[85]
3.	*Scenedesmus obliquus*		Put (1 mmol/L)	CO_2_	Larger biomass production. Affecting the PUT/SPM ratio and influencing the size of LHCII	[86]
4.	*Cucumis sativus cv. Dar*	Drought stress	SPD (1.0 mM)/SPM (1.0 mM)	NO	Reduced tissue damage	[87]
5.	*White clover* (*cv Ladino*)	Polyethylene glycol-induced drought stress	SPD (20µM)	NO (50 µM SNP)	Reduced oxidative damage in leaves by upregulating the genes and activity of antioxidant enzymes via the induction of NO, Ca^2+^, and H_2_O_2_ signaling.	[79]
6.	*Wheat* (*seedlings*)	Drought	Spd (1 mM), 5 mM MGBG (a suppressant of Spd and Spm production via the inhibition of SAM decarboxylase)	50 mM ethephon, an ethylene-releasing agent	ACC, put concentration, and ethylene evolution rate were markedly elevated during severe water deficit. Ethephon causes excessive buildup of Put in grain, and decrease in spd and spm concentrations, which might harm grain filling.	[88]
7.	White clover cultivar ‘Ladino’	Dehydration (by 15% solution of PEG)	Spd (20µM)	NA	Spd-induced H_2_S signaling affects dehydration-regulated antioxidant enzyme activity and gene expression in white clover leaves.	[89]
8.	*Spinacia oleracea*	drought stress treatment for 8 days	NA	100µM NaHS (donor of H_2_S)	Total free and conjugated polyamines (PAs) levels were elevated in the leaves of NaHS-treated plants relative to control plants under comparable drought and re-watering circumstances.	[90]

## Data Availability

Not Applicable.
